# Environmental noise exposure, early biological risk and mental health in nine to ten year old children: a cross-sectional field study

**DOI:** 10.1186/1476-069X-10-39

**Published:** 2011-05-14

**Authors:** Rosanna Crombie, Charlotte Clark, Stephen A Stansfeld

**Affiliations:** 1 Queen Mary, University of London, Barts and the London School of Medicine and Dentistry, Wolfson Institute of Preventive Medicine, Centre for Psychiatry, Room 103, Old Anatomy Building, Charterhouse Square, EC1M 6BQ, London

**Keywords:** Aircraft noise, road traffic noise, low birth weight, premature, psychological distress, mental health, SDQ

## Abstract

**Background:**

Previous research suggests that children born prematurely or with a low birth weight are more vulnerable to the mental health effects of ambient neighbourhood noise; predominantly road and rail noise, at home. This study used data from the Road Traffic and Aircraft Noise Exposure and Children's Cognition and Health (RANCH) study to see if this finding extends to aircraft and road traffic noise at school.

**Methods:**

Children and their parents from schools around three European airports were selected to represent a range of aircraft and road traffic noise exposure levels. Birth weight and gestation period were merged to create a dichotomous variable assessing 'early biological risk'. Mental health was assessed using the Strengths and Difficulties Questionnaire (SDQ). Complete data were available for 1900 primary school children.

**Results:**

Children who were 'at risk' (i.e. low birth weight or premature birth) were rated as having more conduct problems and emotional symptoms and poorer overall mental health than children not at risk. However, there was no interaction between aircraft or road traffic noise exposure at school and early biological risk.

**Conclusions:**

Data from the RANCH study suggests that children with early biological risk are not more vulnerable to the effects of aircraft or road traffic noise at school on mental health than children without this risk; however they are more likely to have mental ill-health.

## Background

Noise pollution levels, particularly from aircraft are expected to increase in the United States of America [1] and it is estimated that in Europe approximately 80 million people are living in areas where the noise levels exceed those recommended [2]. Research regarding noise pollution has shown associations between noise and auditory problems [3] as well as non-auditory health problems [4,5] including mental health [6] and cognitive development [7-9]. Children may be particularly vulnerable to the effects of noise because they may have less cognitive capacity to understand environmental issues and anticipate stressors and they may lack appropriate coping strategies to deal with noise [10,11]. Additionally, noise may interfere with learning at a critical developmental stage [7].

The RANCH study is the largest epidemiological study to date to look at the effects of aircraft and road traffic noise at school on cognition and health in children [4,7,8]. The findings from the RANCH study regarding associations between aircraft and road traffic noise at school and mental health have been reported elsewhere [12]. They showed that aircraft noise at school was associated with increased parent-rated hyperactivity, and unexpectedly that road traffic noise at school was associated with reduced parent-rated conduct problems. Emotional problems, peer problems and prosocial behaviour were not related to noise levels at school.

One particularly important question regarding the relationship between noise and mental health is whether there is a differential pattern of effect within the population. Lercher *et al*. [13] found an interaction between early biological risk and ambient neighbourhood noise (predominantly road and rail noise at home) in children such that children who were born prematurely or were of a low birth weight reported more mental health problems than those without this early biological risk. In their study ambient neighbourhood noise was estimated for the child's home address, however, a large part of a child's day is spent at school where they may also be exposed to environmental noise. It is therefore possible that the moderating effect of early biological risk found by Lercher *et al*. may also exist for the relationship between noise exposure *at school *and mental health.

Lercher *et al*.s [13] measure of ambient neighbourhood noise was calculated by modelling sources of noise from rail, highway and local main roads and then adjusting for Ldn (day-night averaged sound level) measurements taken at certain sites. It is therefore an aggregated measure of environmental noise exposure. The RANCH study has data available for aircraft and road traffic noise at school making it possible to look at the individual contributions of noise from these sources to the effect of early biological risk on mental health.

The aim of this study is to investigate whether early biological risk moderates the relationship between road traffic noise or aircraft noise at school and mental health. To assess this, the model used to analyse the association between aircraft and road traffic noise at school and mental health in the RANCH study [12] will be further adjusted by adding in interaction terms between early biological risk and aircraft noise and road traffic noise at school. We hypothesise that early biological risk will moderate the relationship between both aircraft noise and road traffic noise at school and mental health.

## Methods

### Sampling and Design

Data for this paper were taken from the RANCH project [7,8], a cross-sectional epidemiological field study. Schools around major airports in three European countries (Schiphol in Amsterdam, the Netherlands; Barajas in Madrid, Spain and Heathrow in London, United Kingdom) were classified in terms of noise exposure on a four-by-four grid ranging ordinally from low to high for aircraft noise and low to high for road traffic noise. For each of the 16 noise exposure grid cells, two schools in Spain and the United Kingdom and one school in the Netherlands was selected and, within schools, mixed-ability classes of boys and girls aged nine to ten years were selected to take part. No children were excluded from the selected classes. In total 2279 children and their parents participated in the study from 89 schools. The overall child response rate for the study was 89%. In order to make comparisons between models for the current analyses, subjects with missing values were not included, thus the subsample consisted of 1900 children (Netherlands = 558, Spain = 559, United Kingdom = 783; Males = 897, Females = 1003). There was evidence that participants with missing data were more likely to: have unemployed parents (p < .001), have parents who are not home owners (p < .001), not speak the main language of the country (p < .001), have a mother with lower educational attainment (p < .001), be from an non-crowded home (p < .001).

### Procedure

Children who were selected took part in a two hour testing session one day at school. After this they took home a questionnaire for their main carer to complete. Written consent was obtained from both parents and the children. Ethical approval was obtained in each country [7].

### Measures

#### Mental Health Assessment

Mental Health was measured using the parent-rated version of the Strengths and Difficulties Questionnaire (SDQ) and was included as part of the questionnaire taken home by the child for their main carer. The SDQ is a 25 item behavioural screening questionnaire consisting of five subscales (emotional symptoms, conduct problems, hyperactivity/inattention, peer relationship problems and prosocial behaviour) that can be analysed separately or summed to create a total SDQ score [14]. The total score and three subscales (emotional symptoms, conduct problems and hyperactivity) were analysed in separate models. Scores on the subscales range from zero to ten and scores on the total SDQ scale range from 0-40 (the 5^th ^scale, prosocial behaviour is not included when calculating the total SDQ score), where a higher score indicates poorer mental health. The SDQ can also be split into 'normal', borderline' and 'abnormal' categories using certain cut off points, where children in the abnormal category are classed as 'cases' of psychological distress. This is not a diagnostic tool but can be used to identify children that are likely to have mental health disorders.

#### Early Biological Risk Assessment

Information regarding the child's birth weight and gestation period was obtained through the questionnaire taken home by the child for their main carer. A dichotomous variable 'early biological risk' corresponding to the construct Lercher *et al*. [13] used was created. Participants who were born prematurely (before 36 weeks) or had a low birth weight (under 2500 g) or both were coded as 'at risk' and those who were not in either of these categories were coded as 'no risk'.

#### Road Traffic and Aircraft Noise Exposure Assessment at School

Noise was a continuous measure calculated for each school in dB (A) (A-weighted decibels, where A-weighted means that the sound pressure levels in various frequency bands across the audible range have been weighted in accordance with differences in human hearing sensitivity at different frequencies) where aircraft noise was based on 16-hour outdoor LAeq contours (continuous equivalent sound level of aircraft noise within an area from 07:00 to 23:00 within a specified period) and road traffic noise was derived from modelled data for the Netherlands and from a combination of modelling the proximity to motorways, major roads, and minor roads; traffic flow data; and noise measurements taken at the façade of the school building for the United Kingdom and Spain [15]. In order to reduce the impact of other environmental noise sources, schools which were exposed to dominant sources of noise other than aircraft or road traffic noise (i.e. rail noise) were excluded from the study.

### Sociodemographic variables

The RANCH study collected data on a large number of potential confounding factors. A confounding factor was retained in the analysis if there was a significant relationship between the confounding factor and aircraft noise at school and/or road traffic noise at school (p < .05). Additionally all confounding factors (except main language spoken at home and parental support) were significantly correlated (p < .05) with at least one of the mental health outcomes. Potential confounding factors assessed were: country (United Kingdom (UK), Spain or the Netherlands); gender (male or female); age (measured in days); employment status (highest employment status for household, coded into a dichotomous variable, employed or not employed); crowding at home (the number of people per room in the child's home, coded into a dichotomous variable, crowded or not crowded, according to the cut-off points for each country i.e. one and a half in the United Kingdom and Spain and one in the Netherlands); home ownership (whether the child's home is rented or owned/mortgaged); mother's educational attainment (measured by using a relative inequality index based on a ranked index of standard qualifications in each country [16]; long-standing illness (whether the child is reported by their main carer as having attention deficit hyperactivity disorder, asthma/bronchitis, eczema, epilepsy, depression, diabetes, or dyslexia, coded into a dichotomous variable: has a long standing illness or no long standing illness); main language spoken at home (a dichotomous variable created to indicate whether the child spoke the predominant language for the country at home); parental support for school work (assessed by a self-report scale completed by the child) and classroom glazing type (a measure of the glazing of the windows in the child's classroom, single, double or triple).

### Analysis

Data was analysed using MLwiN multilevel modelling software, where a random intercept model was used to take account of the hierarchical nature of the data, with pupils clustered in schools. Four models were run for each of the mental health outcomes; emotional symptoms, conduct problems, hyperactivity and overall SDQ. Model 1 (unadjusted) contained early biological risk, aircraft and road traffic noise at school; model 2 (adjusted) was the same as Stansfeld *et al*. [12] and included aircraft and road traffic noise at school plus the potential confounding factors: country, gender, age, employment status, crowding at home, home ownership, mother's educational attainment, long-standing illness, main language spoken at home, parental support for school work and classroom glazing type. Model 3 was the same as model 2 but with the addition of early biological risk as a main effect. Model 4 further added an interaction term between noise exposure at school (either aircraft or road traffic noise) and early biological risk. Statistical significance was tested by comparing the goodness of fit of a model with, and without the variable, using a chi-square test of deviance. None of the models were affected significantly by the school level variance (level 2 variance).

## Results

### Sample Characteristics

11.5% of the sample were in the 'biological risk' category (no biological risk = 1704, biological risk = 196). Of those with biological risk 23.5% (n = 46) were premature, 39.8% (n = 78) were born with a low birth weight and the remaining 36.7% (n = 72) were both premature and of a low birth weight. Table 1 illustrates the characteristics of the sample by early biological risk. From this we can see that there is a higher percentage of British and a lower percentage of Dutch among those with early biological risk. Further there are a higher percentage of females, and children with unemployed and non-home owning parents with early biological risk compared to the no early biological risk category. Classroom glazing also varied by early biological risk.

**Table 1:** Sample characteristics by early biological risk

	Not at risk n = 1704	At risk n = 196	p*
Country (%)			

UK	40.3	49.5	

Netherlands	30.2	22.4	

Spain	29.6	28.1	.026

Gender (%)			

Male	48.2	38.3	

Female	51.8	61.7	.008

Age in days			

Mean (SD)	3844.5 (180.4)	3858.8 (192.5)	.298

Employment status (%)			

Unemployed	13.0	20.9	

Employed	87.0	79.1	.002

Crowding at (%)			

Not crowded	79.5	83.7	

Crowded	20.5	16.3	.169

Home ownership (%)			

Not owned	24.6	31.6	

Owned	75.4	68.4	.032

Mother's educational achievement			

Mean (SD)	0.49 (0.28)	0.51 (0.30)	.260

Long-standing illness (%)			

No	76.0	73.0	

Yes	24.0	27.0	.348

Main language spoken at home (%)			

No	8.7	10.7	

Yes	91.3	89.3	.345

Parental support scale			

Mean (SD)	10.0 (2.0)	10.3 (1.8)	.129

Classroom glazing type (%)			

Single	50.4	55.6	

Double (or both single and double)	46.4	44.4	

Triple	3.2	0.0	.024

*p represents significance value for χ^2 ^test or t-test depending on nature of data.

In terms of mental health, 15.5% of the participants were cases on the emotional symptoms subscale (scoring 5+), 14.5% on the conduct problems subscale (scoring 4+) and 16.5% on the hyperactivity subscale (scoring 7+). 10.6% of the sample had a total SDQ score that was abnormal (scoring 18+). The main analyses used the continuous SDQ measures of mental health.

### Sociodemographic variables

The pattern of sociodemographic status predictors was largely similar across models, thus only model 3 with total SDQ score as the outcome variable is presented in Table 2. Country was significantly associated with all the SDQ outcome measures, indicating that children in the UK and the Netherlands were reported to have better mental health than those in Spain. Males were reported to have significantly poorer mental health than females on the total SDQ measure, conduct problems and hyperactivity, but this finding was reversed for the emotional symptoms measure. Non-home ownership, longstanding illness, lower educational achievement of the child's mother, and lower parental support for school work were all significantly associated with all SDQ measures indicating poorer mental health for these children. Somewhat unexpectedly, living in a crowded home was associated with lower reported scores on the hyperactivity subscale, and high levels of classroom glazing were associated with higher reported scores on the conduct problems subscale and the total SDQ score. Age, employment status and main language spoken at home were not significantly associated with any of the SDQ measures. We additionally examined whether length of time the child had attended the school altered the findings of our analyses but it did not, so this variable was not included in the final models.

**Table 2:** Parameter estimates for sociodemographic status variables on the total SDQ score.

	Β	SE	95% CI
Fixed Coefficients			

Spain	1.000		

United Kingdom	-2.131	0.392	-2.899, -1.363*

Netherlands	-3.595	0.408	-4.395, -2.795*

Gender	-0.875	0.249	-1.363, -0.387*

Age	-0.001	0.001	-0.003, 0.001

Employment status	0.430	0.388	-0.330, 1.190

Crowding at home	-0.437	0.326	-1.076, 0.202

Home ownership	1.736	0.315	1.119, 2.353*

Mother's educational achievement	2.400	0.455	1.508, 3.292*

Long-standing illness	1.790	0.290	1.222, 2.358*

Main language spoken at home	-0.239	0.460	-1.141, 0.663

Parental support for school work	-0.306	0.070	-0.443, -0.169*

Classroom glazing type	0.334	0.130	0.079, 0.589*

Random Parameters			

Level 2: school	0.000	0.000	

Level 1: pupil	28.900	0.938	

* p < .05; SE, standard error; CI, confidence interval; SDQ, Strengths and Difficulties Questionnaire.

### Effects of aircraft noise and road traffic noise at school on mental health

Aircraft noise at school ranged from 30-77dB (A). There was a significant effect of aircraft noise at school on hyperactivity after adjustment for country, gender, age, employment status, crowding, home ownership, mother's educational attainment, long-standing illness, main language spoken at home, parental support for school work and classroom glazing type (Table 3: model 2) (χ^2 ^= 3.91, *df *= 1, p < .05) which became borderline significant with further adjustment for early biological risk (Table 3: model 3) (χ^2 ^= 3.72, *df *= 1, *p *= .054) (Table 3). Referring to model 3, this indicates a 0.01 point rise in hyperactivity for every 1dB (A) rise in aircraft noise at school. There were no significant associations between aircraft noise at school and overall SDQ, emotional symptoms and conduct problems, either before or after the addition of early biological risk (Table 3: models 2 and 3). An additional model for hyperactivity was run where children with ADHD were re-classified as having no long-standing illness, but this did not alter the pattern of results.

**Table 3:** Parameter estimates for aircraft and road traffic noise and early biological risk on all SDQ measures

		Model 1 (unadjusted)				Model 2 (adjusted*)				Model 3 (adjusted*)			
		**β**	**SE**	**CI**	**p**	**β**	**SE**	**CI**	**p**	**β**	**SE**	**CI**	**p**

Overall SDQ	Constant	10.07	1.56	7.01, 13.12		14.21	3.40	7.54, 20.88		14.96	3.39	8.31, 21.61	
	
	Aircraft noise	-0.02	0.02	-0.05, 0.02	.35	0.01	0.01	-0.02, 0.04	.39	0.01	0.01	-0.02, 0.04	.51
	
	Road traffic noise	0.00	0.02	-0.04, 0.05	.84	-0.02	0.02	-0.05, 0.01	.27	-0.02	0.02	-0.05, 0.01	.21
	
	Early biological risk	1.81	0.43	0.97, 2.65	.00					1.64	0.41	0.84, 2.45	.00

Emotional symptoms	Constant	2.28	0.47	1.36, 3.19		3.59	1.32	1.00, 6.18		3.82	1.32	1.24, 6.40	
	
	Aircraft noise	-0.00	0.01	-0.01, 0.01	.91	0.00	0.01	-0.01, 0.01	.34	0.00	0.01	-0.01, 0.01	.97
	
	Road traffic noise	0.00	0.01	-0.01, 0.02	.81	0.00	0.01	-0.01, 0.01	.97	-0.00	0.01	-0.01, 0.01	.89
	
	Early biological risk	0.61	0.16	0.30, 0.92	.00					0.52	0.16	0.21, 0.83	.00

Conduct problems	Constant	2.57	0.51	1.57, 3.56		2.27	0.98	0.35, 4.18		2.45	0.98	0.54, 4.36	
	
	Aircraft noise	-0.02	0.01	-0.03, -0.00	.01	-0.01	0.00	-0.01, 0.00	.23	-0.01	0.00	-0.01, 0.00	.17
	
	Road traffic noise	-0.00	0.01	-0.01, 0.01	.91	-0.01	0.01	-0.02, -0.00	.04	-0.01	0.01	-0.02, -0.00	.03
	
	Early biological risk	0.43	0.12	0.19, 0.66	.00					0.40	0.12	0.17, 0.63	.00

Hyperactivity	Constant	3.46	0.71	2.08, 4.85		5.95	1.51	2.99, 8.91		6.02	1.51	3.06, 8.99	
	
	Aircraft noise	-0.01	0.01	-0.02, 0.01	.56	0.01	0.01	0.00, 0.02	.05	0.01	0.01	0.00, 0.02	.05
	
	Road traffic noise	0.01	0.01	-0.01, 0.03	.30	0.00	0.01	-0.01, 0.01	.96	-0.00	0.01	-0.01, 0.01	.94
	
	Early biological risk	0.17	0.19	-0.21, 0.54	.39					0.16	0.18	-0.20, 0.52	.38

* model adjusted for country, age, gender, employment status, crowding, home ownership, mother's educational achievement, long-standing illness, main language spoken at home, parental support for schoolwork and classroom glazing type; SE, standard error; CI, confidence interval; SDQ, strengths and difficulties questionnaire.

Road traffic noise at school ranged from 32-71dB (A). There was a significant effect of road traffic noise at school on conduct problems after adjustment for country, gender, age, employment status, crowding, home ownership, mother's educational attainment, long-standing illness, main language spoken at home, parental support for school work and classroom glazing type (Table 3: model 2) (χ^2 ^= 4.20, *df *= 1, p < .05) which remained after further adjustment for early biological risk (Table 3: model 3) (χ^2 ^= 4.74, *df *= 1, p < .05) (Table 3). Referring to model 3, this revealed a 0.01 point decrease in conduct disorder for every one dB (A) increase in road traffic noise at school. There were no significant associations between road traffic noise at school and overall SDQ, emotional symptoms and hyperactivity, either before or after the addition of early biological risk (Table 3: models 2 and 3).

### Effects of early biological risk on mental health

For early biological risk, the pattern of results was the same in the unadjusted and adjusted models, thus parameters quoted are from the adjusted model (model 3) (Table 3). A significant effect of biological risk was found for the total SDQ score (χ^2 ^= 15.97, *df *= 1, p < .001) where those at risk score on average 1.641 more points than those not at risk. Similarly there was a significant association between early biological risk and emotional symptoms (χ^2 ^= 10.88, *df *= 1, p < .001) with those at risk scoring on average 0.520 points more than those not at risk. There was also a significant association between early biological risk and conduct problems (χ^2 ^= 11.63, *df *= 1, p < .001) with those at risk scoring on average 0.402 points more than those not at risk. Finally there was no significant association between early biological risk and hyperactivity.

### Interaction between early biological risk and noise exposure

Model 4 included an interaction term between early biological risk and either aircraft or road traffic noise exposure at school. There were no significant interactions between early biological risk and aircraft noise at school for any of the outcomes: total SDQ (χ^2 ^= 0.82, *df *= 1, p = .37), emotional symptoms (χ^2 ^= 0.57, *df *= 1, p = .45), conduct problems (χ^2 ^= 0.33, *df *= 1, p = .56), hyperactivity (χ^2 ^= 0.74, *df *= 1, p = .39). Further there were also no significant interactions between early biological risk and road traffic noise at school for any of the outcomes: total SDQ (χ^2 ^= 0.01, *df *= 1, p = .92), emotional symptoms (χ^2 ^= 0.75, *df *= 1, p = .39), conduct problems (χ^2 ^= 1.22, *df *= 1, p = .27), hyperactivity (χ^2 ^= 0.33, *df *= 1, p = .57).

## Discussion

The present study explored the effect of early biological risk as a moderator of the association of noise exposure at school and mental health using data from the RANCH study. Contrary to our hypotheses, no interaction was found between either road traffic or aircraft noise at school and early biological risk for mental health outcomes. Nevertheless a main effect of early biological risk on mental health was found. Further, results regarding noise exposure at school and mental health were similar to Stansfeld *et al*. [12] after adjustment for early biological risk as expected.

The finding of no interaction between early biological risk and either aircraft or road traffic noise at school was unexpected as it does not support Lercher *et al*. [13] who found an interaction between early biological risk and ambient neighbourhood noise in children. Methodological variations between the two studies could account for the difference in findings, such as the noise source measured (aircraft noise and road traffic noise at school versus ambient neighbourhood noise which was predominantly rail and road noise); whether noise was estimated for the school or home environment; the quality of the road exposure assessments, the measurement of mental health (parental report versus self report) and the measurement of early biological risk (parental report versus doctors' entry). Further, the finding of no interaction between aircraft noise at school and early biological risk could be explained by the transient nature of aircraft noise compared to the steady state sound levels emitted by ambient neighbourhood noise.

The finding of a main effect of early biological risk with mental health was of interest. Specifically, participants at risk were rated by their parents as scoring higher on the total SDQ scale and the emotional symptoms and conduct problems subscales, than participants not at risk. These findings support previous studies that have found increased rates of behavioural problems in low birth weight children and higher externalising and internalising symptoms in children who were born prematurely [17-19]. The main effect of early biological risk did not extend to hyperactivity which does not support previous studies that implicate low birth weight in attention deficit hyperactivity disorder (ADHD) [17,19-21] and may be due to the SDQ not adequately capturing hyperactivity as defined in ADHD. Nevertheless, postulated mechanisms for how early biological risk might affect mental health in childhood include increased cell death from postnatal complications [19] or minor neurological abnormalities [22] but more research could be instructive. Despite a lack of understanding in this area, early detection of psychiatric problems can improve the effectiveness of treatment, thus, in the meantime, increased vigilance for children who may be vulnerable to psychiatric problems because of their early biological risk is warranted.

In comparison with Stansfeld *et al*. [12] we found similar main effects of aircraft and road traffic noise on mental health after adjustment for early biological risk; hyperactivity increased with aircraft noise and conduct problems decreased with road traffic noise. The exception was that after the addition of early biological risk, hyperactivity became borderline significant, rather than significantly related to aircraft noise at school. This is likely due to lower power in the current analyses because of the smaller sample used due to missing data with the inclusion of the early biological risk variable. It is thought that noise affects hyperactivity through arousal. According to the arousal theory, noise exposure changes arousal level, which may lead to raised physiological activity levels which might become manifest as psychological difficulties [10]. Such mechanisms are also generally conceptualized as fitting the stress-diathesis model, in which noise exposure increases arousal, and chronic exposure leads to chronic physiological changes and subsequent health effects. However, the finding of reduced conduct problems with increased road traffic noise was unexpected and is not consistent with this theory. Stansfeld *et al*. [12] suggest that this finding may be due to chance, or due to the difficulties in accurately measuring road traffic noise.

The findings of this study must be considered carefully. The cross-sectional nature of the design where selection was based on noise exposure at school and not birth weight/prematurity could have led to an unrepresentative sample, where those with prolonged noise exposure are noise 'survivors', and those that are less resilient have migrated away. The cross-sectional design also does not allow for assessment of potential timing effects of noise exposure. An individual's total noise exposure level is likely to vary beyond the school level measured in the current study, for example, individual daily road traffic noise exposure may vary according to residential road traffic noise exposure (such data was not available for the UK and Spain in the current sample) or an individual's daily school noise exposure may differ due to classroom acoustics. Development of more accurate measures for individual noise exposure as well as longitudinal research would be beneficial for future noise research.

Further limitations of this study include possible biasing introduced from the sample characteristic differences between those with and without early biological risk and between those with and without missing data. Conservative bias is expected from the latter, where those with missing data generally had lower socioeconomic status and thus higher rates of mental health problems. In terms of the measures used, the parent-rated version of the SDQ may underreport internalising disorders [23], however, a self-report version is not available for this age group and may be less reliable [24], thus the parent-rated version represents the most appropriate measure under such circumstances. An additional teacher-rated SDQ would have improved the identification of externalising disorders but was not possible because of teacher burden [23,25]. The early biological risk variable combined information on gestation and birth weight preventing assessment of the individual contributions of each variable, however such variables are likely to be highly related. Further, the early biological risk variable relied upon parental reports. Parents of children with early biological risk may be sensitised to perceive vulnerability in their children which could lead to increased reports of mental health problems for such children. Road traffic is a source of both noise pollution and air pollution which have both been linked to mental health outcomes [12,26]. This study did not adjust for the effects of air pollution which may act as a confounder or have a synergistic effect when coupled with noise [27]. Despite these limitations this study benefits from the fact that a range of noise levels were investigated and that the findings adjusted for multiple sociodemographic status factors.

## Conclusions

Data from the RANCH study suggests that children with early biological risk; that is those born prematurely or with a low birth weight, have a greater chance of developing certain mental health outcomes but are not more vulnerable to the effects of aircraft and road traffic noise at school on mental health. This highlights the need to develop understanding of the pathways through which early biological risk might operate. The results must be considered carefully due to potential bias from the cross-sectional design used and sample characteristic differences between those with and without early biological risk. Nevertheless, this study tested a wide range of noise exposure levels and adjusted for many potential confounding factors.

## List of abbreviations

RANCH: Road Traffic and Aircraft Noise Exposure and Children's Cognition and Health; SDQ: Strengths and Difficulties Questionnaire; UK: United Kingdom; ADHD: Attention Deficit Hyperactivity Disorder.

## Competing interests

The authors declare that they have no competing interests.

## Authors' contributions

RC carried out analysis and interpretation of data, drafted and edited the manuscript. CC contributed to the conception and design, the acquisition of data, the analysis and interpretation of data and manuscript revisions. SS contributed to the conception and design, the acquisition and interpretation of data and manuscript revisions. All authors have given final approval of the version to be published.

## References

[B1] Financial Aviation Administration Performance and Accountability Report 2009 Washington, U.S. Department of Transport 1 150

[B2] Commission of the European Communities Future noise policy, European Commission, Green Paper 1996 Brussels, European Commission 1 31

[B3] Kryter KD The effects of noise on man 1985 2 Orlando, Florida: Academic Press

[B4] Clark C Stansfeld SA The effect of transportation noise on health and cognitive development: A review of recent evidence Int J Comp Psychol 2007 20 145 158

[B5] Stansfeld S Haines M Brown B Noise and health in the urban environment Rev Environ Health 2000 15 43 82 10.1515/REVEH.2000.15.1-2.43 10939085

[B6] Hardoy MC Carta MG Marci AT Carbone F Cadeddu M Kovess V Dell'Osso L Carpiniello B Exposure to aircraft noise and risk of psychiatric disorders: The Elmas survey Social Psychiatry and Psychiatric Epidemiology 2005 40 24 26 10.1007/s00127-005-0837-x 15624071

[B7] Stansfeld SA Berglund B Clark C Lopez-Barrio I Fischer P Öhrström E Haines MM Head J Hygge S van Kamp I Berry BF Aircraft and road traffic noise and children's cognition and health: a cross-national study Lancet 2005 365 1942 1949 10.1016/S0140-6736(05)66660-3 15936421

[B8] Clark C Martin R van Kempen E Alfred T Head J Davies HW Haines MM Lopez Barrio I Matheson M Stansfeld SA Exposure-effect relations between aircraft and road traffic noise exposure at school and reading comprehension: the RANCH project Am J Epidemiol 2006 163 27 37 16306314 10.1093/aje/kwj001

[B9] Hygge S Evans GW Bullinger M A prospective study of some effects of aircraft noise on cognitive performance in school children Psychological Science 2002 13 469 474 10.1111/1467-9280.00483 12219816

[B10] Cohen S Evans GW Stokols D Krantz DS Behavior, health and environmental stress 1986 New York: Plenum Press

[B11] Evans GW Kielwer W Martin J Schroeder HE The role of the physical environment in the health and well-being of children New Directions in Health Psychology Assessment. Series in Applied Psychology: Social Issues and Questions 1991 New York: Hemisphere Publishing Corp 127 157

[B12] Stansfeld SA Clark C Cameron RM Alfred T Head J Haines MM van Kamp I van Kempen E Lopez-Barrio I Aircraft and road traffic noise exposure and children's mental health Journal of Environmental Psychology 2009 29 203 207 10.1016/j.jenvp.2009.01.002

[B13] Lercher P Evans GW Meis M Kofler WW Ambient neighbourhood noise and children's mental health Occup Environ Med 2002 59 380 386 10.1136/oem.59.6.380 12040113 PMC1740306

[B14] Goodman R The Strengths and Difficulties Questionnaire: a research note Journal of Child Psychology & Psychiatry 1997 38 581 586 10.1111/j.1469-7610.1997.tb01545.x 9255702

[B15] Department of Transport and the Welsh Office Calculation of road traffic noise (CRTN) 1998 London, HMSO

[B16] Mackenbach JP Kunst AE Measuring the magnitude of socio-economic inequalities in health: An overview of available measures illustrated with two examples from Europe Soc Sci Med 1997 44 757 771 10.1016/S0277-9536(96)00073-1 9080560

[B17] Elgen I Sommerfelt K Markestad T Population based, controlled study of behavioural problems and psychiatric disorders in low birthweight children at 11 years of age Arch Dis Child Fetal Neonatal Ed 2002 87 F128 132 10.1136/fn.87.2.F128 12193521 PMC1721443

[B18] Middle C Johnson A Alderdice F Petty T Macfarlane A Birthweight and health and development at the age of 7 years Child: Care, Health and Development 2002 22 55 71 8640964

[B19] Bhutta AT Cleves MA Casey PH Cradock MM Anand KJ Cognitive and behavioral outcomes of school-aged children who were born preterm: a meta-analysis JAMA 2002 288 728 737 10.1001/jama.288.6.728 12169077

[B20] Stein RE Siegel MJ Bauman LJ Are children of moderately low birth weight at increased risk for poor health? A new look at an old question Pediatrics 2006 118 217 223 10.1542/peds.2005-2836 16818568

[B21] Botting N Powls A Cooke RW Marlow N Attention deficit hyperactivity disorders and other psychiatric outcomes in very low birthweight children at 12 years J Child Psychol Psychiatry 1997 38 931 941 10.1111/j.1469-7610.1997.tb01612.x 9413793

[B22] Breslau N Chilcoat HD Johnson EO Andreski P Lucia VC Neurologic soft signs and low birthweight: their association and neuropsychiatric implications Biol Psychiatry 2000 47 71 79 10.1016/S0006-3223(99)00131-6 10650451

[B23] Goodman R Ford T Simmons H Gatward R Meltzer H Using the Strengths and Difficulties Questionnaire (SDQ) to screen for child psychiatric disorders in a community sample British Journal of Psychiatry 2000 177 534 539 10.1192/bjp.177.6.534 11102329

[B24] Muris P Meesters C Eijkelenboom A Vincken M The self-report version of the Strengths and Difficulties Questionnaire: Its psychometric properties in 8- to 13-year-old non-clinical children Br J Clin Psychol 2004 43 437 448 10.1348/0144665042388982 15530213

[B25] Goodman R Ford T Simmons H Gatward R Meltzer H Using the Strengths and Difficulties Questionnaire (SDQ) to screen for child psychiatric disorders in a community sample International Review of Psychiatry 2003 15 166 172 10.1080/0954026021000046128 12745328

[B26] Lundberg A Psychiatric aspects of air pollution Otolaryngol Head Neck Surg 1996 114 227 231 10.1016/S0194-5998(96)70172-9 8637739

[B27] Davies HW Vlaanderen JJ Henderson SB Brauer M Correlation between co-exposures to noise and air pollution from traffic sources Occup Environ Med 2009 66 347 350 10.1136/oem.2008.041764 19017692

